# Use of mild cognitive impairment and prodromal AD/MCI due to AD in clinical care: a European survey

**DOI:** 10.1186/s13195-019-0525-9

**Published:** 2019-08-22

**Authors:** Daniela Bertens, Stephanie Vos, Patrick Kehoe, Henrike Wolf, Flavio Nobili, Alexandre Mendonça, Ineke van Rossum, Jacub Hort, Jose Luis Molinuevo, Michael Heneka, Ron Petersen, Philip Scheltens, Pieter Jelle Visser

**Affiliations:** 1 Alzheimer Centre, Department of Neurology, VU University Medical Centre, De Boelelaan 1118, 1081 HZ Amsterdam, The Netherlands; 2 Alzheimer Centre, School for Mental Health and Neuroscience (MHeNS), Maastricht University, Maastricht, The Netherlands; 3 Learning and Research, Faculty of Health Sciences, University of Bristol, Bristol, UK; 4 Department of Psychiatry, University of Zurich, Zürich, Switzerland; 5 Clinical Neurology Unit, Department of Neuroscience (DINOGMI), University of Genoa, Genoa, Italy; 6 Department of Neurology and Laboratory of Neurosciences, Faculty of Medicine, University of Lisbon, Lisbon, Portugal; 7 Department of Neurology, 2nd Faculty of Medicine Charles University in Prague and Motol University Hospital, Prague, Czech Republic; 8 BarcelonaBeta Brain Research Center, Pasqual Maragall Foundation, Barcelona, Spain; 9 Alzheimer’s Disease and Other Cognitive Disorders Unit, Neurology Service, Hospital Clínic, Barcelona, Spain; 10 Clinical Neuroscience, Department of Neurology Clinical Neuroscience Unit, and German Center for Neurodegenerative Disease (DZNE), Bonn, Germany; 11 Mayo Clinic Alzheimer’s Disease Research Center and the Mayo Clinic Study of Aging, Rochester, MN USA

**Keywords:** Survey, Questionnaire, MCI, Prodromal AD, MCI due to AD

## Abstract

**Introduction:**

The diagnosis of mild cognitive impairment (MCI) refers to cognitive impairment not meeting dementia criteria. A survey among members of the American Association of Neurology (AAN) showed that MCI was considered a useful diagnosis. Recently, research criteria have been proposed for the diagnosis of Alzheimer’s disease (AD) in MCI based on AD biomarkers (prodromal AD/MCI due to AD). The aim of this study was to investigate the attitudes of clinicians in Europe on the clinical utility of MCI and prodromal AD/MCI due to AD criteria. We also investigated whether the prodromal AD/MCI due to AD criteria impacted management of MCI patients.

**Methods:**

An online survey was performed in 2015 among 102 members of the European Academy of Neurology (EAN) and the European Alzheimer’s Disease Consortium (EADC). Questions were asked on how often criteria were used, how they were operationalized, how they changed patient management, and what were considered advantages and limitations of MCI and prodromal AD/MCI due to AD. The questionnaire consisted of 47 questions scored on a Likert scale.

**Results:**

Almost all respondents (92%) used the MCI diagnosis in clinical practice. Over 80% of the EAN/EADC respondents found a MCI diagnosis useful because it helped to label the cognitive problem, involve patients in planning for the future, and start risk reduction activities. These findings were similar to those reported in the AAN survey. Research criteria for prodromal AD/MCI due to AD were used by 68% of the EAN/EADC respondents. The most common reasons to use the criteria were increased certainty of diagnosis (86%), increased possibilities to provide counseling (51%), facilitation of follow-up planning (48%), start of medical intervention (49%), and response to patients’ wish for a diagnosis (41%). Over 70% of the physicians considered that a diagnosis of prodromal AD/MCI due to AD had an added value over the MCI diagnosis.

**Conclusions:**

The diagnostic criteria of MCI and prodromal AD/MCI due to AD are commonly used among EAN/EADC members. The prodromal AD/MCI due to AD were considered clinically useful and impacted patient management and communication.

**Electronic supplementary material:**

The online version of this article (10.1186/s13195-019-0525-9) contains supplementary material, which is available to authorized users.

## Introduction

The term mild cognitive impairment (MCI) was originally developed for research in non-demented patients with objective memory impairment. These subjects were assumed to be at an increased risk of developing dementia, in particular Alzheimer-type dementia [1–4]. A survey among members of the American Academy of Neurology (AAN) showed that MCI was a common diagnosis and considered useful [5]. However, little is known about the clinical use of MCI outside Northern America, where attitudes towards MCI may be different. Moreover, since the introduction of the MCI concept, research criteria have been proposed to diagnose Alzheimer’s disease (AD) among subjects with MCI, referred to as prodromal AD and MCI due to AD [6, 7]. These criteria require, in addition to a diagnosis of MCI, the presence of biomarkers indicative for AD pathophysiology. Research criteria for prodromal AD were proposed by an International Working Group (IWG), and criteria for MCI due to AD by the National Institute on Aging–Alzheimer’s Association (NIA-AA). According to the IWG-1 criteria, prodromal AD is defined by abnormal amyloid, tau, or FDG-PET or by hippocampal atrophy, while according to IWG-2 criteria, prodromal AD is defined by abnormal beta amyloid 1–42 and tau in cerebrospinal fluid (CSF) or an abnormal amyloid PET scan [7, 8]. NIA-AA MCI due to AD is diagnosed with high likelihood in the presence of both abnormal amyloid and neuronal injury markers, and with intermediate likelihood, in case only an amyloid or injury marker is measured and this marker is abnormal [6]. Although these criteria are intended for research, they are also used in clinical practice [9]. However, it is unknown how often these criteria are used, how they are implemented, what is communicated to patients, how it affects the management of patients, and what are considered advantages and disadvantages of the criteria relative to the MCI diagnosis. Information on how MCI and the research criteria are perceived and used is critical for the development and implementation of guidelines for MCI in clinical practice, and to develop procedures to enable international standardization.

The survey on MCI among members AAN was performed in 2010. It showed that the term MCI was frequently used. Perceived benefits of the use of MCI included that the diagnosis facilitated planning for the future, motivated risk reduction activities, and financial planning. Drawbacks for using the term MCI were the difficulty to diagnose it and the fact that a diagnosis could cause unnecessary worry and that MCI could better be described as early Alzheimer’s disease (AD). Despite these critics, the benefits were thought to outweigh the drawbacks.

The aim of the present study was first to apply the AAN questionnaire to clinicians in Europe. The survey assessed the frequency of the use of the MCI diagnosis, implications for communication with and management of patients meeting criteria, and perceived strengths and limitations of the MCI concept. Secondly, we assessed the attitude towards the use of the preclinical AD/MCI due to AD criteria. We asked on the frequency of use of these criteria, how they were operationalized, what were reasons to use the criteria or not in clinical practice, whether a diagnosis of prodromal AD/MCI due to AD impacted patient management compared to MCI patient that did not meet prodromal AD/MCI due to AD criteria, and whether they provided added value over the MCI diagnosis without biomarkers.

## Methods

We conducted a European multicenter survey regarding the usefulness of MCI and prodromal AD/MCI due to AD in clinical practice (see Additional file 1), based on an adapted version of the MCI survey of Roberts and colleagues [5].

### Survey development and contents

The first part of the survey was on MCI with sections on “terms and definitions,” “current practice,” and “attitudes” towards the use of the criteria in clinical practice. This part consisted of 26 questions scored on a Likert scale. It was the same as the AAN questionnaire with a few changes. We added questions on diagnostic investigations performed in patients with MCI and counseling on alcohol intake. We also rephrased the question on the disease codings according to European classification systems. In the second part of the survey, we explored attitudes towards criteria for prodromal AD/MCI due to AD. The format of the questions was similar to part 1, except that we added 2 questions on possible benefits (selection patients for trials, planning for follow-up) and deleted the statement “MCI is usually better described as early AD.” A steering group with both European and American representatives evaluated the survey content and the appropriateness of adjustments made. We pilot tested the survey in our center to check how much time it would take and if there were any suggestions. Next, we formatted the survey such that it could be administered online using the Bristol Online Survey system (https://www.onlinesurveys.ac.uk). The final version of the questionnaire consisted of 47 questions (16 from Roberts in part 1, and 31 in part 2), and it took 15–20 min to complete it.

### Participant identification and recruitment

We invited members of the European Academy of Neurology (EAN) and European Alzheimer’s Disease Consortium (EADC). The EAN unites neurologists across Europe and has members from 45 European national neurological societies and includes 800 individual neurologists (https://www.eaneurology.org/). The EADC is a network of over 50 European centers that have a strong track record of clinical and biomedical research in AD and related dementias (http://www.eadc.info/sito/pagine/home.php).

We sent a link to the survey to members of the EAN scientific panel on Dementia and Cognitive Disorders (*n* = 74) and to EADC members (*n* = 140). We encouraged EAN and EADC members to share the survey to other colleagues working in the dementia field but who were not necessarily EAN or EADC members. We send our first mail on 28 November 2014 and reminders on 12 and 19 December 2014. In addition, EAN members were invited to complete the survey via a web link that was provided in the November 2014 EAN newsblog, which is sent to over 15,000 members of national neurological societies in Europe. The survey was live between November 2014 and January 2015.

### Analysis

Responses to survey items and demographics were expressed as percentages. We tested differences in responses between the EAN/EADC and the AAN respondents regarding the use of the MCI diagnosis. In a subgroup of respondents that used the diagnosis of “Prodromal AD” or “MCI due to AD” in clinical practice, we tested whether terminology, counseling, and management differed between patients with a diagnosis of prodromal AD/MCI due to AD and those without that diagnosis. Group differences were tested by the chi-square test. If a question had multiple responses, we used the chi-square test for trend. A two-sided *p* value < 0.05 was considered statistically significant.

## Results

### Respondents

One hundred and two completed surveys were obtained, of which 38 from EADC members (response rate 27%), 53 from the EAN (including 16 members of the Spanish Association of Neurology), 3 from members of the International Psychogeriatric Association (IPA), and 8 from other or unknown sources. Demographic characteristics of the respondents are shown in Table 1.

**Table 1:** Demographics

	EAN/EADC	AAN^^^
Age (mean (SD))*	50.0 (8.7)	54 (8.6)
Gender, *n* female/total (%)	26/84 (31%)	21%
Medical specialty, *n* (%)		
- Neurology	74 (73%)	94.4%
- Geriatrics	9 (8.8%)	3.1%
- Psychiatry	9 (8.8%)	3.4%
- Neuropsychology	2 (1.9%)	4.4%
- Combination of the above	7 (6.9%)	NA
Subspecialty training, *n* yes (%)	65 (63.7%)	59.6%
Years of experience, *n* (%)		NA
- < 5 years	7 (6.9%)	
- 5–10 years	15 (14.7%)	
- 10–15 years	20 (19.6%)	
- 15–20 years	20 (19.6%)	
- > 20 years	40 (39.2%)	
Organization, *n* (%)		
- Solo practice	5 (4.9%)	30%
- Group practice	57 (59.9%)	48.4%
Single specialty group	1 (1.0%)	
Multispecialty group	6 (5.9%)	
University-based group	50 (49%)	
- Hospital or clinic	38 (37.3%)	17.9%
Health care center	2 (2.0%)	
Government hospital or clinic	28 (27.5%)	
Other public or private hospital or clinical setting	8 (7.8%)	
- Other	2 (2.0%)	3.6%
Number of respondents within a country, *n*		NA
- Turkey, Denmark, Finland, Georgia, Ireland, Israel, Luxembourg, Poland	1	
- Austria, Belgium, Bulgaria, Norway, Slovenia, Portugal	2	
- Sweden, Greece, Romania, Netherlands, France, Italy, UK	3–6	
- Czech Republic, Germany	7–9	
- Spain	33	

*Abbreviation*: *NA* not available

*Response from 75 subjects

^^^Only percentages reported

### MCI

#### Terms and definitions of clinical diagnosis

Ninety-two percent of the respondents recognized MCI as a clinical diagnosis. Of these, 85% differentiated between amnestic versus non-amnestic MCI forms and 65% used the subdivision of single versus multiple domain MCI. A minority of respondents used cognitive impairment no dementia (CIND; 16%), age-associated memory impairment (AAMI; 13%), or other terms (8%).

#### Clinical practice

Ninety-three percent of the respondents saw patients with cognitive symptoms of mild severity routinely in practice. A small proportion (6%) saw them once or twice per month and 1% never. The most common terms to describe the cognitive impairment to patients were MCI (75%), memory problems (46%), and possibly early AD (45%).

**Fig. 1:**
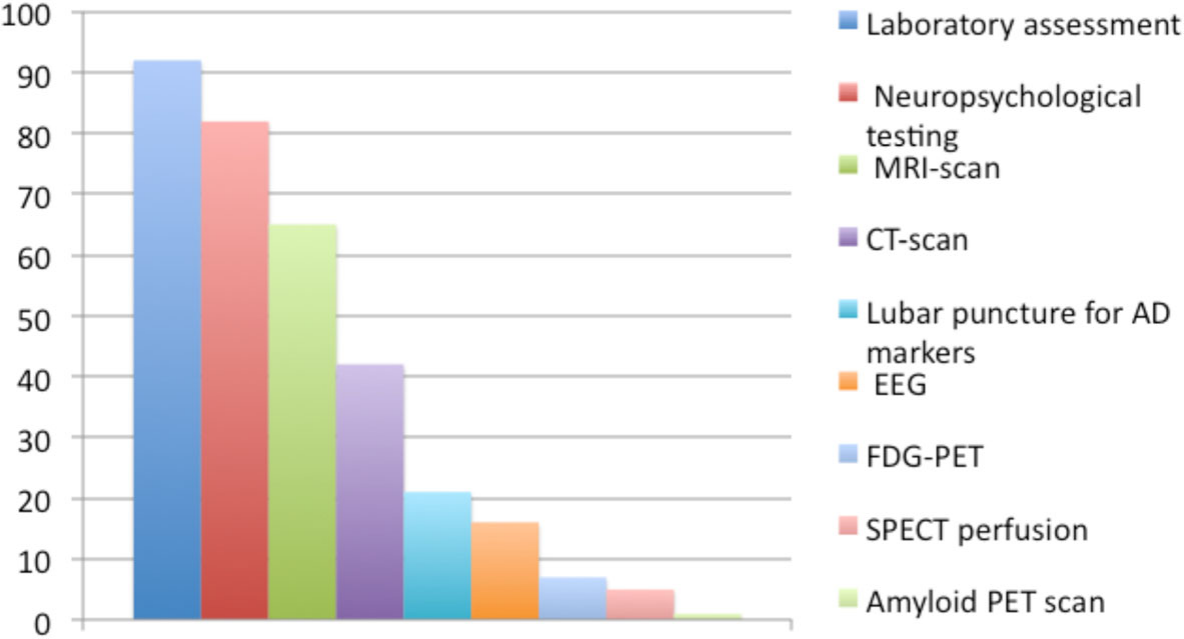
Diagnostic investigations routinely performed in patients with MCI. Bars indicate frequencies (in %). Abbreviations: AD Alzheimer’s disease, CT computed tomography, MRI magnetic resonance imaging, EEG electroencephalogram, SPECT single-photon emission computed tomography, FDG-PET fludeoxyglucose positron emission tomography

Laboratory assessment was performed routinely in 92% of patients with MCI, neuropsychological testing in 82%, magnetic resonance imaging (MRI) in 65%, and computed tomography in 42%. Less than 25% of respondents performed routinely lumbar puncture, electroencephalography (EEG), fludeoxyglucose positron emission tomography (FDG-PET), single-photon emission computed tomography (SPECT), or amyloid PET (Fig. 1).

**Fig. 2:**
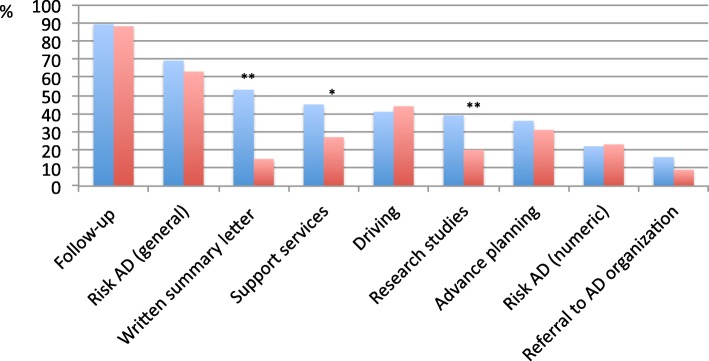
Topics that are routinely discussed with patients with MCI. Blue bars are frequencies (in %) of respondents of EAN/EADC who discuss routinely the above topics. Red bars are frequencies (in %) of respondents of the AAN survey. Abbreviations: EAN European Academy of Neurology, EADC European Alzheimer’s Disease Consortium, AAN American Academy of Neurology. Difference frequencies between EAN/EADC and AAN members were tested using chi-square. *p < 0.01, **p < 0.0001

Respondents routinely advised patients on mental and physical exercise. Less than 50% of the respondents discussed routinely diet and nutrition, alcohol intake use of vitamins, and vascular risk factors (Table 2 and Additional file 2: Figure S1). Most respondents routinely discussed with patients the need for follow-up (89%) and the risk of AD in general terms (69%). Less than 40% of the respondents discussed routinely numeric estimates of AD, considerations in relation to driving, potential to undertake research studies, support service options available, potential benefits of advance planning, and referral to the Alzheimer Association or similar organizations (Table 2 and Fig. 2). A small proportion of the respondents routinely prescribed cholinesterase inhibitors (21%), memantine (13%), or other medications (e.g., vitamins or supplements, or antidepressants) (Table 2 and Additional file 2: Figure S1).

**Table 2:** Usual practice when seeing patients with cognitive symptoms of mild severity

		Never	Rarely	Sometimes	Routinely	*p* value*
Patient counseling						
- Diet and nutrition	EAN/EADC	9 (8.8)	11 (10.8)	32 (31.4)	49 (48)	0.17
	AAN	57 (13.6)	69 (16.4)	132 (31.5)	162 (38.5)	
- Vitamins	EAN/EADC	16 (15.7)	24 (23.5)	38 (37.3)	23 (22.5)	0.29
	AAN	49 (11.6)	84 (20.1)	155 (36.9)	132 (31.4)	
- Mental exercise	EAN/EADC	1 (1)	4 (3.9)	13 (12.7)	83 (81.4)	0.41
	AAN	13 (3.0)	21 (5.1)	71 (17.0)	315 (74.9)	
- Physical exercise	EAN/EADC	3 (2.9)	4 (3.9)	13 (12.7)	82 (80.4)	0.78
	AAN	8 (1.8)	19 (4.5)	67 (15.9)	327 (77.8)	
- Alcohol	EAN/EADC	18 (17.6)	14 (13.7)	32 (31.4)	36 (35.3)	**
Patient education						
- Advance planning	EAN/EADC	1 (1)	21 (20.6)	42 (41.2)	37 (36.3)	0.08
	AAN	33 (7.9)	84 (20.0)	172 (41.0)	130 (31)	
- Driving	EAN/EADC	3 (2.9)	16 (15.7)	42 (41.2)	41 (40.2)	0.82
	AAN	11 (2.6)	52 (12.3)	186 (44.2)	172 (40.9)	
- Research studies	EAN/EADC	2 (2)	12 (11.8)	47 (46.1)	40 (39.2)	< 0.0001*
	AAN	27 (6.4)	109 (26)	199 (47.4)	85 (20.2)	
- Support services	EAN/EADC	0	17 (16.7)	37 (36.3)	46 (45.1)	0.001*
	AAN	18 (4.3)	92 (21.9)	195 (46.4)	115 (27.3)	
- Recommendations for follow-up	EAN/EADC	0	1(1)	8 (7.8)	91 (89.2)	0.75
	AAN	0	5 (1.3)	44 (10.4)	371 (88.3)	
- Risk of AD and related disorder (general terms)	EAN/EADC	1 (1)	5 (4.9)	25 (24.5)	70 (68.6)	0.55
	AAN	4 (1)	37 (8.9)	113 (27)	265 (63)	
- Risk of AD and related disorders (numeric estimates)	EAN/EADC	7 (6.9)	24 (23.5)	47 (46.1)	22 (21.6)	0.17
	AAN	47 (11.1)	125 (29.8)	150 (35.7)	98 (23.4)	
- Referral to Alzheimer’s association or similar association	EAN/EADC	15 (14.7)	34 (33.3)	35 (34.3)	16 (15.7)	0.22
	AAN	82 (19.5)	153 (36.5)	146 (34.7)	39 (9.3)	
- Written summary letter of findings for patient	EAN/EADC	9 (8.8)	13 (12.7)	25 (24.5)	54 (52.9)	< 0.0001*
	AAN	117 (27.8)	158 (37.6)	82 (19.6)	63 (14.9)	
Medication prescribed						
- Cholinesterase inhibitors	EAN/EADC	28 (27.5)	16 (15.7)	35 (4.3)	21 (20.6)	0.01*
	AAN	60 (14.3)	67 (15.9)	189 (45)	104 (24.8)	
- Memantine	EAN/EADC	64 (62.7)	14 (13.7)	7 (6.9)	13 (12.7)	0.0001*
	AAN	147 (35.1)	108 (25.6)	129 (30.7)	36 (8.5)	
- Other	EAN/EADC	26 (25.5)	7 (6.9)	28 (27.5)	9 (8.8)	0.89
	AAN	140 (33.3)	45 (10.8)	169 (40.2)	66 (15.7)	

All data are *N* (%)

*Abbreviations*: *EAN* European Academy of Neurology, *EADC* European Alzheimer’s Disease Consortium, *AAN* American Academy of Neurology

*Differences in replies between EAN/EADC and AAN members (chi-square)

**Question not in the AAN survey

#### Attitudes towards MCI

More than 80% of the respondents agreed strongly or somewhat with the statement that labeling cognitive problems as MCI was helpful for patients and family members and that labeling helps for future planning and helps to engage in risk reduction. Furthermore, more than 50% of the respondents agreed strongly or somewhat with the statement that labeling aids planning of insurance and finances and that certain medications can be helpful for some patients. More than 50% of the respondents disagreed strongly or somewhat with the statements that MCI is usually better described as AD, that diagnosing causes unnecessary worry for patients and family members, that it is too difficult to diagnose MCI accurately or reliably, and that it makes no sense to diagnose it because there is no approved treatment. In a free text section inviting open comment, the most common response was that MCI is a broad symptomatic diagnosis without a description of the etiology and needed further investigation (Table 3 and Additional file 2: Figure S2).

**Table 3:** Perceptions of benefits, drawbacks, and limitations of MCI as a clinical diagnosis

Benefits		Strongly agree	Somewhat agree	Neither agree or disagree	Somewhat disagree	Strongly disagree	*p* value
- Labeling the problem is helpful for patients and family members	EAN/EADC	52 (51)	38 (37.3)	7 (6.9)	2 (2)	3 (2.9)	0.12
	AAN	191 (45.5)	192 (45.7)	24 (5.6)	11 (2.7)	2 (0.5)	
- A diagnosis is useful so the patient can be more involved in planning for the future	EAN/EADC	44 (43.1)	41 (40.2)	8 (7.8)	5 (4.9)	4 (3.9)	0.15
	AAN	184 (43.8)	180 (42.8)	37 (8.8)	16 (3.9)	3(0.7)	
- A diagnosis can be useful in motivating the patient to engage in risk reduction activities	EAN/EADC	43 (42.2)	42 (41.2)	9 (8.8)	6 (5.9)	2 (2)	0.61
	AAN	148 (35.2)	207 (49.4)	39 (9.3)	21 (4.9)	5 (1.2)	
- A diagnosis helps the family with insurance planning	EAN/EADC	23 (22.5)	30 (29.4)	32 (31.4)	9 (8.8)	8 (7.8)	0.18
	AAN	90 (21.5)	143 (34.1)	143 (34.1)	31 (7.3)	12 (2.9)	
- A diagnosis helps the family with financial planning	EAN/EADC	25 (24.5)	41 (40.2)	27 (26.5)	4 (3.9)	5 (4.9)	0.35
	AAN	121(28.7)	183 (43.6)	93 (22.1)	15 (3.6)	8 (1.9)	
- Certain medications can be useful for treating some patients	EAN/EADC	16 (15.7)	39 (38.2)	18 (17.6)	16 (15.7)	13 (12.7)	0.034
	AAN	76 (18.0)	199 (47.3)	75 (17.8)	49 (11.7)	21 (5.1)	
Drawbacks and limitations							
- Diagnosing causes unnecessary worry for patients and family members	EAN/EADC	8 (7.8)	12 (11.8)	11 (10.8)	28 (27.5)	43 (42.2)	0.0001*
	AAN	8 (2.0)	74 (17.6)	75 (17.8)	155 (36.8)	109 (25.9)	
- There is no approved treatment so it does not make sense to diagnose	EAN/EADC	6 (5.9)	0	6 (5.9)	25 (24.5)	65 (63.7)	0.02*
	AAN	11 (2.7)	24 (5.6)	33 (7.8)	130 (31.0)	222 (52.9)	
- It is too difficult to diagnose accurately or reliably	EAN/EADC	4 (3.9)	10 (9.8)	9 (8.8)	35 (34.3)	44 (43.1)	0.013*
	AAN	8 (2.0)	88 (21.0)	53 (12.7)	147 (34.9)	124 (29.5)	
- MCI is usually better described as early Alzheimer’s disease	EAN/EADC	8 (7.8)	13 (12.7)	23 (22.5)	30 (29.4)	28 (27.5)	0.91
	AAN	26 (6.1)	60 (14.4)	87 (20.8)	138 (32.8)	109 (25.9)	

All data are *N* (%)

*Abbreviations*: *EAN* European Academy of Neurology, *EADC* European Alzheimer’s Disease Consortium, *AAN* American Academy of Neurology

*Differences between answers between EAN/EADC and AAN members were tested using chi-square and are indicated with a *p* value

#### Comparison with results from the AAN survey

Age and gender were similar between AAN and EAN/EADC respondents. Compared to AAN respondents, EAN/EADC respondents were less often neurologist (73% versus 94%) and worked less often in a solo practice (5% versus 30%) and more often in a hospital setting (37% versus 18% (Table 1)). EAN/EADC respondents more often saw patients with cognitive symptoms of mild severity, discussed research studies, and provided a written summary letter of the findings than AAN respondents. EAN/EADC respondents less commonly prescribed cholinesterase inhibitors and memantine, and they less often agreed to the statement that a diagnosis “causes unnecessary worry to patients and family” (*p* < 0.0001 for all comparisons, Tables 2 and 3).

### Prodromal AD/MCI due to AD

#### Research criteria and clinical practice

Sixty-eight percent of the respondents used the research criteria for diagnosing prodromal AD/MCI due to AD (22% used the IWG criteria, 35% the NIA-AA criteria, and 43% used both). Those who used the research criteria did not differ in demographics or membership of EADC or EAN, compared to non-users.

Reasons for the use of the research criteria were increased certainty of diagnosis in almost 90%. Around 50% of the respondents used the research criteria to increase possibilities to provide counseling and facilitation of follow-up planning and to start medical intervention. Less than 50% of the respondents used the criteria to respond to patients’ wish for a diagnosis and to facilitate selection for clinical trial selection or observational studies.

Thirty-two percent of the respondents did not use the research criteria. The most common reasons were lack of standardized measurements and cut-off values (42%), lack of treatment possibilities (42%), and lack of implication on a single-level case (33%). In less than 30% of the respondents, there was an inability to perform a biomarker measurement, a concern that the diagnosis might upset patients and their family; the lack of inclusion in national guidelines, or the lack of added value in diagnosing prodromal AD/MCI due to AD over a diagnosis of MCI.

Of the criteria users, 49% applied the research criteria only in a subset. This group applied criteria if they perceived a clinical need (38%) or if the patient wished to know (26%). Other reasons indicated in the open text field, were younger age, and selection of subjects for research. Seventy percent of the respondents always disclosed the diagnosis. The most common reason (50%) not to disclose diagnosis was the wish of patients not to know. Other reasons not to disclose the diagnosis were diagnostic uncertainty and fear that the diagnosis might upset patients or their family. When the diagnosis was not disclosed, information was still used to plan future care by 75%.

**Fig. 3:**
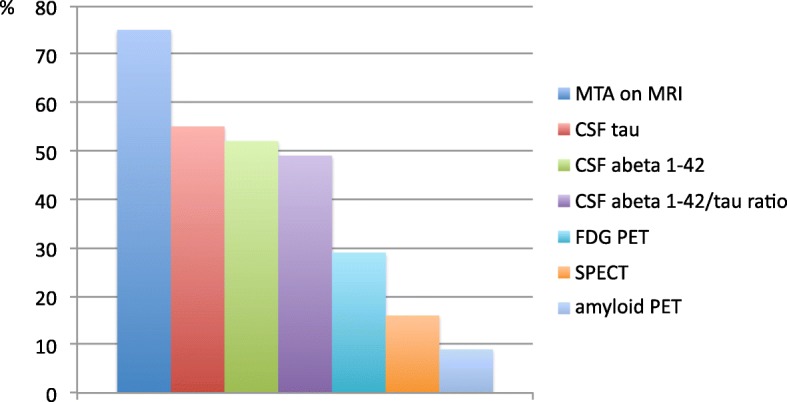
Use of biomarkers for scoring the research criteria. Bars indicate responders (in %) that often or always performed diagnostic assessment of the above-mentioned tests. Abbreviations: MTA medial temporal atrophy, MRI magnetic resonance imaging, CSF cerebrospinal fluid, abeta amyloid beta, FDG-PET fludeoxyglucose positron emission tomography, SPECT single-photon emission computed tomography, PET positron emission tomography

Biomarkers always or often used to define prodromal AD/MCI due to AD were MRI measures of the medial temporal atrophy (MTA, 75%), CSF amyloid beta 1–42, or tau (52–55%). Less than 30% used FDG-PET, SPECT, or amyloid PET (Fig. 3).

The use of research criteria appeared to influence the communication towards patients. The term “MCI” was less often used and terminology including “possible early AD” more often compared to the use of MCI as diagnostic label (*p* = 0.001). When patients met the research criteria, respondents more often used the term “possible early AD” and “early AD” (*p* < 0.0001) and less often patients were told they had “memory problems or difficulties” or “did not have dementia or AD” compared to subjects not meeting the criteria (*p* < 0.0001).

Counseling by respondents on diet and nutrition, vitamins, mental exercise, physical exercise, and alcohol did not differ between patients with or without criteria for prodromal AD/MCI due to AD. However, patients who met the criteria for prodromal AD/MCI due to AD respondents were more often counseled on the need for follow-up, risk for AD in general terms advanced planning, driving, participation in research studies, availability of support services, and access to other support organizations and were more often prescribed cholinesterase inhibitors compared to patients who did not meet the criteria (Table 4 and Additional file 2: Figure S3).

**Table 4:** Usual practice when seeing patients with or without prodromal AD/MCI due to AD

		Never	Rarely	Sometimes	Routinely	*p* value*
Patient counseling						
- Diet and nutrition	Prodromal AD	7 (11)	5 (8)	25 (38)	28 (43)	0.97
	No prodromal AD	8 (12)	6 (9)	24 (37)	27 (42)	
- Vitamins	Prodromal AD	15 (23)	19 (29)	23 (35)	8 (12)	0.99
	No prodromal AD	14 (22)	19 (29)	24 (37)	8 (12)	
- Mental exercise	Prodromal AD	2 (3)	2 (3)	7 (10)	56 (84)	0.45
	No prodromal AD	3 (4)	5 (8)	10 (15)	48 (73)	
- Physical exercise	Prodromal AD	1 (1)	1 (1)	10 (15)	55 (82)	0.31
	No prodromal AD	1 (2)	4 (6)	15 (23)	46 (69)	
- Alcohol	Prodromal AD	10 (15)	13 (20)	22 (33)	21 (32)	0.95
	No prodromal AD	8 (13)	12 (19)	24 (38)	20 (30)	
Patient education						
- Advance planning	Prodromal AD	0	6 (9)	28 (41)	34 (50)	0.0001*
	No prodromal AD	6 (9)	20 (30)	26 (39)	14 (21)	
- Driving	Prodromal AD	0	5 (7)	27 (40)	36 (53)	0.0001*
	No prodromal AD	5 (8)	18 (27)	28 (42)	15 (23)	
- Research studies	Prodromal AD	0	5 (7)	32 (47)	31 (46)	0.0001*
	No prodromal AD	6 (9)	25 (38)	26 (39)	9 (14)	
- Support services	Prodromal AD	0	11 (16)	24 (36)	32 (48)	0.003*
	No prodromal AD	4 (6)	25 (38)	19 (29)	18 (27)	
- Recommendations for follow-up	Prodromal AD	0	2 (3)	4 (6)	61 (91)	0.0001*
	No prodromal AD	0	10 (15)	15 (22)	42 (63)	
- Risk of AD and related disorder (general terms)	Prodromal AD	0	1 (2)	23 (34)	42 (64)	0.0001*
	No prodromal AD	3 (5)	17 (27)	22 (34)	22 (34)	
- Risk of related disorders (numeric estimates)	Prodromal AD	9 (13)	19 (28)	27 (40)	12 (18)	0.33
	No prodromal AD	13 (20)	24 (38)	19 (30)	8 (12)	
- Referral to Alzheimer’s association or similar organization	Prodromal AD	6 (9)	16 (24)	30 (46)	14 (21)	0.0001*
	No prodromal AD	29 (45)	20 (31)	12 (18)	4 (6)	
- Written summary letter of findings for patient and family	Prodromal AD	6 (9)	6 (9)	18 (27)	37 (55)	0.91
	No prodromal AD	6 (9)	8 (12)	19 (29)	33 (50)	
Medication prescribed						
- Cholinesterase inhibitors	Prodromal AD	9 (13)	8 (12)	26 (39)	24 (36)	0.0001*
	No prodromal AD	40 (61)	18 (27)	6 (9)	2 (3)	
- Memantine	Prodromal AD	44 (66)	13 (19)	7 (11)	3 (4)	0.30
	No prodromal AD	52 (79)	10 (15)	3 (4)	1 (2)	
- Other	Prodromal AD	12 (32)	5 (13)	16 (42)	5 (13)	0.46
	No prodromal AD	13 (37)	2 (6)	18 (51)	2 (6)	

All data are *N* (%)

*Abbreviation*: *AD* Alzheimer’s disease

*Differences in replies between patients meeting criteria for prodromal AD/MCI due to AD and patients not meeting criteria for prodromal AD/MCI due to AD (chi-square)

#### Attitudes towards prodromal AD/MCI due to AD

In the total group, over 80% of the respondents agreed strongly or somewhat to the statement that a diagnosis of prodromal AD/MCI due to AD was helpful for labeling the problem for patients and family members, was helpful for inclusion into clinical trials, aided physicians to plan for follow-up, involved patients in future planning, and motivated patients to engage in risk reduction activities. It was also frequently reported (62–76%) that a diagnosis could help family and patients with financial or insurance planning and could help in the decision to start medication.

**Fig. 4:**
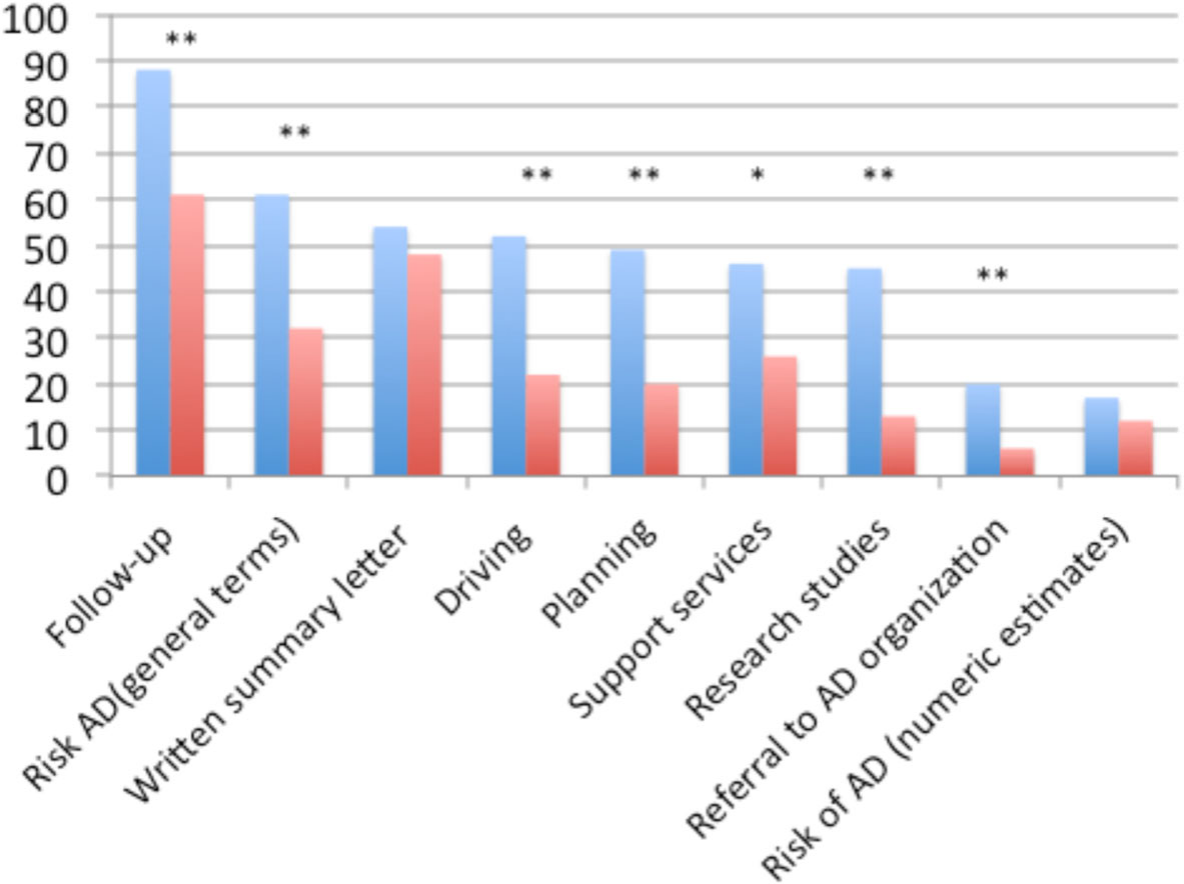
Topics of discussion in patients with and without prodromal AD. Frequency (%) of respondents that routinely discussed the above topics in patients with “prodromal AD” (blue column) or “no prodromal AD” (red column). Difference frequencies between “prodromal AD” and “no prodromal AD” were tested using chi-square. *p < 0.01, **p < 0.0001

The majority of all respondents strongly or somewhat disagreed with the statement that diagnosing prodromal AD/MCI due to AD has no added value over a diagnosis of MCI (70%). There was also considerable disagreement with the suggestions that it is too difficult to diagnose it accurately or reliably (69%), that it causes unnecessary worry for patients and family members (69%), and that there is no approved treatment so it does not make sense to diagnose it (73%) (Table 5 and Fig. 4).

**Table 5:** Perceptions of benefits, drawbacks, and limitations of prodromal AD/MCI due to AD in clinical practice

	Strongly agree	Somewhat agree	Neither agree or disagree	Somewhat disagree	Strongly disagree
Benefits					
- Labeling the problem is helpful for patients and family members	53 (52)	33 (32)	6 (6)	4 (4)	6 (6)
- A diagnosis is useful so the patient can be more involved in planning for the future	45 (44)	38 (37)	10 (10)	6 (6)	3 (3)
- A diagnosis can be useful in motivating the patient to engage in risk reduction activities	43 (42)	40 (39)	10 (10)	5 (5)	4 (4)
- A diagnosis helps the family with insurance planning	28 (27)	35 (34)	27 (26)	6 (6)	6 (6)
- A diagnosis helps the family with financial planning	30 (29)	47 (46)	16 (16)	4 (4)	5 (5)
- A diagnosis can be useful for including patients in clinical trials	71 (70)	23 (23)	5 (5)	3 (3)	0
- Certain medications can be useful for treating some patients	26 (25)	39 (38)	13 (13)	10 (10)	14 (14)
- A diagnosis is useful for the physician to plan the follow-up	58 (57)	29 (28)	7 (7)	5 (5)	3 (3)
Drawbacks and limitations					
- Diagnosing causes unnecessary worry for patients and family members	4 (4)	14 (14)	14 (14)	36 (35)	34 (33)
- There is no approved treatment so it does not make sense to diagnose	13 (13)	3 (3)	12 (12)	23 (23)	51 (50)
- It is too difficult to diagnose accurately or reliably	4 (4)	21 (21)	7 (7)	30 (30)	40 (39)
- A diagnosis has no added value over the diagnosis of MCI	8 (8)	9 (9)	14 (14)	21 (21)	50 (49)

All data are *N* (%)

*Abbreviation*: *MCI* mild cognitive impairment

## Discussion

According to this survey, the term MCI was widely used and considered to represent a useful clinical entity. MCI definitions, communication with patients, management, and attitudes towards the concept were, with a few exceptions, similar to that of AAN members in a previous study. The novel research criteria for prodromal AD/MCI due to AD were used by 68% of the respondents, of whom 51% used the criteria in all subjects with MCI. The use of the research criteria influenced the communication with and management of subjects with MCI.

MCI was widely used as a clinical diagnosis and also referred to in communications with patients and their family. To diagnose MCI, respondents performed neuropsychological testing, while laboratory assessments, as well as MRI or CT imaging, were performed in the majority of the cases, which is in agreement with the EAN guidelines for the assessment of AD [10]. Respondents perceived that labeling the problem outweighed any negative effect but still 20% agreed with the statement that MCI would cause unnecessary worry for patients and family. Other reported benefits were a better planning for future and motivation of patients to engage risk reduction. Remarkably, prescription of cholinesterase inhibitors, memantine, vitamins, and supplements was relatively common, although there is no evidence of efficacy for these treatments in MCI.

The responses of the EAN/EADC members were generally similar to those of AAN members, despite the time interval of the studies. A major difference was that EAN/EADC respondents less frequently prescribed cholinesterase inhibitors and memantine, compared to AAN respondents. Since such prescription of dementia medications in subjects with MCI is off-label use both in Europe and the USA, AAN members might be more convinced that these medications are of benefit.

Despite being research criteria, the majority of respondents used prodromal AD/MCI due to AD criteria in clinical practice. The main reason given for this use was that the criteria increased the certainty of AD diagnosis. Only 55% often or routinely used an amyloid marker indicating that, in the majority of the cases, an injury marker was used for diagnosis. This is consistent with the NIA-AA criteria of intermediate likelihood and the IWG-1 criteria, but not with the IWG-2 criteria, as these consider amyloid markers as a core diagnostic feature. Yet, IWG-2 criteria were published only a few months before we performed our survey and therefore were unlikely to have been incorporated into clinical practice. The use of either an amyloid or injury marker may result in heterogeneity among subjects with “prodromal AD/MCI due to AD” as there is only moderate overlap between amyloid and injury markers since they become abnormal in different stages of the disease and are supposed to reflect disease processes [11]. Not using amyloid markers may result from lack of awareness of the different diagnostic properties of amyloid and injury markers, lack of training of physicians to perform a lumbar puncture, or lack of possibilities to perform cerebrospinal assessment or amyloid imaging. Moreover, assessment of amyloid pathology is often not mentioned in national guidelines on dementia in Europe nor is it reimbursed. At the time of our survey in 2014, another survey showed that of the 19 countries that were included in our study only 8 had national guidelines that discussed CSF analysis in the assessment of cognitive disorders [12]. CSF analysis was reimbursed in 11 of the 19 countries. Forty-nine percent of the respondents who applied the research criteria did assessments in a subset of patients depending on the clinical need and the wishes of patients. The latter criterion for selection underlines the importance for shared decision-making, since, in light of the lack of treatment possibilities, it is important to inform patients on the benefits and possible drawbacks of making a diagnosis [9]. In line with this, 15% of criteria users did not always disclose diagnosis if the patient or family members did not want to know.

Using research criteria had a major influence on the way patients were communicated with and their management, such as prescription of cholinesterase inhibitors. Despite of the fact that the criteria are meant for research use, respondents apparently found them clinically helpful. Irrespective of the application of the research criteria, all respondents perceived more benefits than drawbacks using the criteria in clinical practice.

This is in accordance with a previously performed EADC survey where respondents claimed to perceive high diagnostic confidence using AD biomarkers and clinical studies that use of amyloid biomarkers increased diagnostic confidence and impacted management [9, 13, 14].

In 2018, the NIA-AA consortium published an update to the NIA-AA criteria, referred to as a research framework [15]. It extends the NIA-AA criteria by basing the diagnosis of AD on amyloid, tau, and neuronal injury markers (ATN staging). Although conceptually the framework is similar to the NIA-AA and IWG-2 criteria as it defines amyloid pathology as necessary for the diagnosis of AD, the combination of both tau and injury markers may be more difficult to implement in clinical practice as the number of diagnostic categories increases.

A limitation of this comparative study was that the EAN/EADC survey was administered 5 years after the AAN survey. Nevertheless, the results were comparable across the two surveys but we cannot exclude the possibility that over time the attitudes towards MCI have changed among AAN members. Another possible limitation was the EAN/EADC survey was held among clinicians with a special interest in neurodegenerative disorders and mainly working at university hospitals, such that the responses may not be wholly generalizable to the broader community of clinicians. We sent our survey to members of the EADC and the EAN scientific panel on Dementia and Cognitive Disorders, which have a research interest in dementing disorders, which limits the generalizability of the findings. Because EADC and EAN members overlap and because we distributed the survey through the EAN newsletter, which is received also by neurologists not working in the dementia field, no response rate could be calculated. As we expanded upon the original AAN survey on MCI by including a second part focusing on the research criteria, this increased the length of the survey and may have increased levels of non-response. Another limitation that may argue against representativeness is that 30% of our respondents were from Spain. However, post hoc analysis showed only a few differences between respondents from Spain compared to other respondents. Finally, questionnaires measured opinions and actual use of the criteria may be different. Our survey highlights that both the MCI and prodromal AD/MCI due to AD criteria are considered clinically useful. Our survey indicated several barriers for the use of the criteria in clinical practice such as lack of standardized measurements and cut-off values of biomarkers [6–8, 15]. This will become even more problematic with the NIA-AA research framework as more biomarkers now can be used for the diagnosis, for which often no established cut-points are available, such as CSF neurofilament light. Moreover, there was variability in type of diagnostic tests used and patient management between clinicians. These findings highlight the need for an update of national and international guidelines on the diagnosis and management of predementia AD and communication to patients. This should take into account cultural background, the age, religious background, and education of patients [16].

In our study, we focused on the opinion of clinicians; however, it will be crucial to assess the opinions and needs of patients in future studies as well.

## Conclusions

Our survey showed that both the MCI and prodromal AD/MCI due to AD criteria are considered clinically useful and that the diagnosis influenced the management of patients meeting these criteria. However, clinicians differed in the tests used to make the diagnosis and in the management of the patients. This highlights the need for standardized national and international guidelines for the diagnosis and management of predementia AD.

## Additional files

Additional file 1: Mild cognitive impairment survey. (PDF 384 kb)

Additional file 2: **Figure S1.** Counseling and medication prescription in patients with MCI. **Figure S2.** Benefits and drawbacks of MCI as clinical diagnosis. **Figure S3.** Counseling and medication prescription in patients with and without prodromal AD. (DOCX 572 kb)

## Data Availability

The datasets analyzed during the current study are available from the corresponding author on reasonable request.

## Abbreviations

AAMI: Age-associated memory impairment
AAN: American Academy of Neurology
AD: Alzheimer’s disease
CIND: Cognitive impairment no dementia
CSF: Cerebrospinal fluid
CT: Computed tomography
EADC: European Alzheimer’s Disease Consortium
EAN: European Academy of Neurology
EEG: Electroencephalography
FDG-PET: Fludeoxyglucose positron emission tomography
IWG: International Working Group
MCI: Mild cognitive impairment
MRI: Magnetic resonance imaging
MTA: Medial temporal atrophy
NIA-AA: National Institute on Aging–Alzheimer’s Association
SPECT: Single-photon emission computed tomography

## References

[1] Gauthier S, Reisberg B, Zaudig M (2006). Mild cognitive impairment. Lancet..

[2] Ganguli M, Dodge HH, Shen C, DeKosky ST (2004). Mild cognitive impairment, amnestic type: an epidemiologic study. Neurology..

[3] Visser PJ (2006). Mild cognitive impairment subtypes and vascular dementia. J Am Geriatr Soc.

[4] Petersen RC, Caracciolo B, Brayne C, Gauthier S, Jelic V, Fratiglioni L (2014). Mild cognitive impairment: a concept in evolution. J Intern Med.

[5] Roberts JS, Karlawish JH, Uhlmann WR, Petersen RC, Green RC (2010). Mild cognitive impairment in clinical care: a survey of American Academy of Neurology members. Neurol Int.

[6] Albert MS, DeKosky ST, Dickson D (2011). The diagnosis of mild cognitive impairment due to Alzheimer’s disease: recommendations from the National Institute on Aging-Alzheimer’s Association workgroups on diagnostic guidelines for Alzheimer’s disease. Alzheimers Dement.

[7] Dubois B, Feldman HH, Jacova C (2014). Position paper advancing research diagnostic criteria for Alzheimer’s disease: the IWG-2 criteria. Lancet Neurol.

[8] Dubois B, Feldman HH, Jacova C (2010). Revising the definition of Alzheimer’s disease: a new lexicon. Lancet Neurol.

[9] Bocchetta M, Galluzzi S, Kehoe PG (2015). The use of biomarkers for the etiologic diagnosis of MCI in Europe: an EADC survey. Alzheimers Dement.

[10] Hort J, O’Brien JT, Gainotti G (2010). EFNS guidelines for the diagnosis and management of Alzheimer’s disease. Eur J Neurol.

[11] Van Rossum IA, Visser PJ, Knol DL (2012). Injury markers but not amyloid markers are associated with rapid progression from mild cognitive impairment to dementia in Alzheimer’s disease. J Alzheimers Dis.

[12] Miller AM, Balasa M, Blennow K (2017). Current approaches and clinician attitudes to the use of cerebrospinal fluid biomarkers in diagnostic evaluation of dementia in Europe. J Alzheimers Dis.

[13] Duits FH, Prins ND, Lemstra AW (2015). Diagnostic impact of CSF biomarkers for Alzheimer’s disease in a tertiary memory clinic. Alzheimers Dement.

[14] Rabinovici GD, Gatsonis C, Apgar C (2019). Association of amyloid positron emission tomography with subsequent change in clinical management among Medicare beneficiaries with mild cognitive impairment or dementia. JAMA.

[15] Jack CR, Bennett DA, Blennow K (2018). NIA-AA research framework: toward a biological definition of Alzheimer’s disease. Alzheimers Dement..

[16] Visser PJ, Wolf H, Frisoni G, Gertz H-J (2012). Disclosure of Alzheimer’s disease biomarker status in subjects with mild cognitive impairment. Biomark Med.

