# Supplementary material for: Use of mild cognitive impairment and prodromal AD/MCI due to AD in clinical care: a European survey
Source: Alzheimers Res Ther. 2019 Aug 22;11:74. doi: 10.1186/s13195-019-0525-9 (PMC6706888; doi:10.1186/s13195-019-0525-9)
Supplement: Supplementary file 1 — Mild cognitive impairment survey. (PDF 384 kb) [file 13195_2019_525_MOESM1_ESM.pdf]

Mild Cognitive Impairment Survey

Edit this page

Welcome

Welcome the online questionnaire on attitudes towards MCI and prodromal AD. The questionnaire has 47 questions divided over 7 sections.

Sections I-III concern MCI as a clinical syndrome and is a copy of the questionnaire performed in the USA by the American Association of Neurology.

Sections IV-VI concern 'prodromal AD/MCI due to AD' and is a new extension. Some questions of sections I-III are repeated in sections IV-VI but now in relation to prodromal AD/MCI due to AD. Even if you do not use the concept of prodromal AD/MCI due to AD in clinical practice please complete section VI.

In section VII you will be asked to provide demographic information.

It will take about 15-20 minutes to complete the questionnaire.
All data collected in this survey will be held anonymously and securely. No personal data is asked for or retained.

Cookies, personal data stored by your Web browser, are not used in this survey.
Note that once you have clicked on the CONTINUE button your answers are submitted and you can not return to review or amend that page.

Mild Cognitive Impairment Survey

Edit this page

Part I. Terms and Conditions: Mild Cognitive Impairment (MCI) as a clinical concept

Note that once you have clicked on the CONTINUE button your answers are submitted and you can not return to review or amend that page.

We recognize that specific definitions of the Mild Cognitive Impairment (MCI) concept vary, and that some use terms other than MCI to describe this concept.

1. Which of the following terms do you recognize as a clinical diagnosis (as opposed to research category)? *(select all that apply)*

- ☐ Cognitive Impairment, No Dementia (CIND)
- ☐ Age-Associated Memory Impairment (AAMI)
- ☐ Mild Cognitive Impairment (MCI)
- ☐ Other *(please specify)*:

If you checked MCI, which subtypes do you use? *(select all that apply)*

- ☐ Amnestic vs. non-amnestic
- ☐ Single vs. multiple domain

2. Please describe the definition(s) you use in clinical practice for the term(s) you endorsed above:

3. Comments to questions 1 - 2

Mild Cognitive Impairment Survey

Edit this page

Part II. Current Practices: Mild Cognitive Impairment (MCI) as a clinical concept

The following questions regard clinical diagnosis of cognitive symptoms of mild severity

. How often do you see patients with **cognitive symptoms of mild severity**?

- Never (Skip to section III, question 15 on page 4)
- Rarely (less than once a month)
- Sometimes (once or twice a month)
- Routinely (several times per month)

. What medical code(s) have you used for diagnostic and/or billing purposes when evaluating a patient with **cognitive symptoms of mild severity**?
select all that apply)

- Alzheimer's disease "ICD-10" (G30.0, G30.1, F00.0, F00.1)

- [ ] Amnestic disorder "DSM IV": NOS (294.8)
- [ ] Cognitive disorder "DSM IV": NOS (294.9)
- [ ] Dementia "ICD-9": NOS (294.8)
- [ ] Memory Loss "ICD-9" (780.93)
- [ ] None
- [ ] Other *(please specify)*:

How often do you perform the following diagnostic investigations in patients with **cognitive symptoms of mild severity**?

	Never	Rarely	Sometimes	Routinely
**a.** Neuropsychological testing	○	○	○	○
**b.** Laboratory assessment (routine blood screen)	○	○	○	○
for AD biomarkers	○	○	○	○
**d.** CT scan	○	○	○	○
**e.** MRI scan	○	○	○	○
**f.** EEG	○	○	○	○
**g.** SPECT perfusion scan	○	○	○	○
**h.** FDG-PET scan	○	○	○	○
**i.** Amyloid-PET scan	○	○	○	○

When communicating to patients with **cognitive symptoms of mild severity** and their family members, how do you describe the patient's cognitive difficulties?
elect all that apply)

- ☐ Memory problems or difficulties
- ☐ Mild cognitive impairment
- ☐ Possible early Alzheimer's disease (AD)
- ☐ Possible early dementia
- ☐ Tell patient/family it's not dementia or AD
- ☐ Other *(please specify)*:

. How often do you counsel patients with **cognitive symptoms of mild severity** on the following issues?

	Never	Rarely	Sometimes	Routinely
**a.** Diet and nutrition	○	○	○	○
**b.** Vitamins or supplements	○	○	○	○
**c.** Mental exercise	○	○	○	○
**d.** Physical exercise	○	○	○	○
**e.** Alcohol	○	○	○	○
**f.** Other	○	○	○	○

. *If other issue, please specify:*

. When you meet with patients with **cognitive symptoms of mild severity** and/or their family members, how frequently does your communication include the following?

	Never	Rarely	Sometimes	Routinely
**a.** Discussion of advance planning				
**b.** Discussion of driving				
**c.** Information on research studies				
**d.** Information on support services				
**e.** Recommendations for monitoring, follow-up				
**f.** Risk of Alzheimer's disease and related disorders (in general terms)				
**g.** Risk of Alzheimer's disease and related disorders (using numeric estimates)				
**h.** Referral to Alzheimer's Association or similar organisation				
**i.** Written summary letter of findings for patient and family				
**j.** Other				

. *If other communication, please specify:*

2. How often do you prescribe the following medications for your patients with **cognitive symptoms of mild severity**?

	Never	Rarely	Sometimes	Routinely

**a.** Cholinesterase inhibitors	○	○	○	○
**b.** Memantine	○	○	○	○
**c.** Other	○	○	○	○

3. *If other medication, please specify:*

14. Comments to question 4-13

Mild Cognitive Impairment Survey

Edit this page

Part III. Attitudes towards MCI as a clinical concept

One way to categorize symptoms of mild severity is the concept of Mild Cognitive Impairment (MCI). Below are statements regarding the potential benefits and limitations of MCI as a clinical diagnosis.

5. Rate the extent to which you agree or disagree with each statement.

	Strongly agree	Somewhat agree	Neither agree or disagree	Somewhat disagree	Strongly disagree
**a.** Labelling the problem is helpful for patients and family members	○	○	○	○	○
**b.** Diagnosing MCI causes unnecessary worry for patients and family members	○	○	○	○	○
**c.** A diagnosis is useful so the patient can be more involved in planning for	○	○	○	○	○

the future					
**d.** A diagnosis helps the family with insurance planning	○	○	○	○	○
**e.** A diagnosis helps the family with financial planning	○	○	○	○	○
**f.** A diagnosis can be useful in motivating the patient to engage in risk reduction activities	○	○	○	○	○
**g.** MCI is usually better described as early Alzheimer's disease	○	○	○	○	○
**h.** Certain medications can be useful for treating some patients with MCI	○	○	○	○	○
**i.** There is no approved treatment for MCI so it does not make sense to diagnose it	○	○	○	○	○
**j.** MCI is too difficult to diagnose accurately or reliably	○	○	○	○	○

6. Please comment further on the benefits and/or limitations of MCI as a clinical diagnosis:

Mild Cognitive Impairment Survey

Edit this page

Part IV. Terms and Conditions: Prodromal Alzheimer's Disease and MCI due to AD

Recently research criteria have been published for prodromal Alzheimer's disease (AD) by an International Working group (IWG-1, Dubois 2007/2010) and for MCI due to AD by the National Institute of Aging/Alzheimer Association (NIA-AA, Albert 2011)

The following questions regard terms and conditions of 'prodromal AD or MCI due to AD' as defined by these criteria.

17. Are you using these criteria in clinical practice?

No (Continue with question 32, section VI on page 7)

Yes

18. Which set of criteria do you use in clinical practice?

IWG prodromal AD criteria

NIA-AA MCI due to AD criteria

Both

9. Which biomarkers do you use for scoring the criteria in clinical practice?

	**Type of biomarker used for the diagnosis of Prodromal AD/MCI due to AD**			
	**Never**	**Sometimes**	**Often**	**Always**
**a.** Medial temporal atrophy on MRI				
**b.** CSF Abeta 1-42				
**c.** CSF t-tau				
**d.** CSF abeta 1-42/tau ratio				
**e.** Amyloid binding on PET				
**f.** Hypometabolism on FDG-PET				
**g.** Hypoperfusion on SPECT				

Part V. Current Practices: Prodromal AD and MCI due to AD

The following questions apply to the use of the criteria of prodromal AD or MCI due to AD criteria in routine clinical practice.

0. How often do you make a diagnosis of prodromal AD or MCI due to AD?

- Never (Continue with question 32, section VI on page 7)
- Rarely (less than once a month)
- Sometimes (every month)
- Routinely (every week)

1. Do you perform an assessment for prodromal AD or MCI due to AD in all patients with MCI or in a subset?

- All
- Subset

If you perform an assessment in a subset only, how do you select subjects for this assessment? *(select all that apply)*

- Wish of patient
- Clinical need
- Other *(please specify)*:

2. Do you always disclose the diagnosis of prodromal AD/MCI due to AD to the patient and caregiver?

- Yes, I always disclose the diagnosis

No, I sometimes do not disclose the diagnosis

i. If you do not disclose always disclose the diagnosis, what is the reason that you do not disclose it?

i. If you do not disclose the diagnosis, do you nevertheless use this information for planning of future care?

Yes

No

3. How do you describe the diagnosis to the patient and their family members when a patient meets criteria for prodromal AD/MCI due to AD? And when a patient does **not** meet the criteria? *Select all that apply*.

	Diagnosis prodromal AD/MCI due to AD	Diagnosis no prodromal AD/MCI due to AD
**a.** Memory problems or difficulties	☐	☐
**b.** Mild cognitive impairment	☐	☐
**c.** Possible early Alzheimer's disease (AD)	☐	☐
**d.** Early Alzheimer's disease	☐	☐
**e.** Possible early dementia	☐	☐
**f.** Tell patient/family it's not dementia or AD	☐	☐
**g.** Other	☐	☐

4. *If other please specify:*

5. How often do you counsel the patient on the following issues when a patient meets criteria for prodromal AD/MCI due to AD? And when a patient does **not** meet the criteria?

	Diagnosis prodromal AD/MCI due to AD	Diagnosis no prodromal AD/MCI due to AD
**a.** Diet and nutrition		
**b.** Vitamins or supplements		
**c.** Mental exercise		
**d.** Physical exercise		
**e.** Alcohol		
**f.** Other		

6. *If other issue, please specify:*

7. How frequently does your communication with the patient and/or family include the following when a patient meets criteria for prodromal AD/MCI due to AD? And when a patient does **not** meet the criteria?

	Diagnosis prodromal AD/MCI due to AD	Diagnosis no prodromal AD/MCI due to AD

**a.** Discussion of advance planning		
**b.** Discussion of driving		
**c.** Information on research studies		
**d.** Information on support services		
**e.** Recommendations for monitoring, follow-up		
**f.** Risk of Alzheimer's disease and related disorders (in general terms)		
**g.** Risk of Alzheimer's disease and related disorders (using numeric estimates)		
**h.** Referral to Alzheimer's Association of similar organisation		
**i.** Written summary letter of findings for patient and family		
**j.** Other		

[illegible]8. *If other communication, please specify:*

[illegible]9. How often do you prescribe the following medications when a patient meets criteria for prodromal AD/MCI due to AD? And when a patient does **not** meet the criteria?

	**Diagnosis prodromal AD/ MCI due to AD**	**Diagnosis no prodromal AD/ MCI due to AD**
**a.** Cholinesterase inhibitors		
**b.** Memantine		
**c.** Other		

[illegible]0. *If other medication, please specify*

[illegible]1. General comments on Current Practices prodromal AD or MCI due to AD:

Mild Cognitive Impairment Survey

Edit this page

Part VI. Attitudes towards prodromal AD and MCI due to AD

*. Below are statements regarding the potential benefits and limitations of prodromal AD or MCI due to AD as a clinical diagnosis. Rate the extent to which you agree or disagree with each statement.

	Strongly agree	Somewhat agree	Neither agree or disagree	Somewhat disagree	Strongly disagree
**a.** Labelling the problem is helpful for patients and family members	○	○	○	○	○
**b.** A diagnosis causes unnecessary worry for patients and family members	○	○	○	○	○
**c.** A diagnosis is useful so the patient can be more involved in planning for the future	○	○	○	○	○
**d.** A diagnosis helps the family with insurance planning	○	○	○	○	○
**e.** A diagnosis helps the family with financial planning	○	○	○	○	○
**f.** A diagnosis can be useful in motivating the patient to engage in risk reduction activities	○	○	○	○	○
**g.** Certain medications can be useful for treating some patients with prodromal AD or MCI due to AD	○	○	○	○	○

**h.** There is no approved treatment for prodromal AD or MCI due to AD so it does not make sense to diagnose it	○	○	○	○	○
**i.** A diagnosis can be useful for including patients in clinical trials	○	○	○	○	○
**j.** Prodromal AD or MCI due to AD is too difficult to diagnose accurately or reliably	○	○	○	○	○
**k.** A diagnosis of prodromal AD or MCI due to AD has no added value over the diagnosis of MCI	○	○	○	○	○
**l.** A diagnosis is useful for the physician to plan the follow-up	○	○	○	○	○

[illegible]. Please comment further on the benefits and/or limitations of prodromal AD/MCI due to AD as a clinical diagnosis:

4. If you apply the criteria of prodromal AD/MCI due to AD in clinical practice, what are the main reasons to do so:

	One of the primary reasons	One of the secondary reasons	Not relevant
**a.** Increase certainty of diagnosis	○	○	○
**b.** Increase counselling opportunities	○	○	○
**c.** To plan follow-up	○	○	○

**d.** To start medical intervention	○	○	○
**e.** The patient has asked for it	○	○	○
**f.** To select subjects for clinical trials	○	○	○
**g.** To select subjects for observational studies	○	○	○
**h.** Other	○	○	○

5. *If other reason, please specify:*

6. If you do **NOT** apply the criteria of prodromal AD/MCI due to AD in clinical practice, what are the main reasons to do so:

	One of the primary reasons	One of the secondary reasons	Not relevant
**a.** Not included in national guidelines	○	○	○
**b.** No added value over a diagnosis of MCI	○	○	○
**c.** No implications for prognosis on a single level case	○	○	○
**d.** No implications for treatment	○	○	○
**e.** Disclosure of diagnosis of prodromal AD may upset a patient	○	○	○
**f.** Lack or standardised measurements and cut-off values biomarkers	○	○	○
**g.** No possibilities to perform biomarker measurements	○	○	○

**h.** Other	○	○	○

7. *If other reason please specify:*

8. Which set of criteria do you prefer: IWG-1 prodromal AD or NIA-AA MCI due to AD and why?

Continue >

Survey testing only

Check Answers & Continue >

Edit this page

Mild Cognitive Impairment Survey

Edit this page

Part VII: Demographic Information

39. In which country do you work?

40. What is your medical speciality?
(select all that apply)

- ☐ Neurology
- ☐ Geriatrics
- ☐ Psychiatry
- ☐ Neuropsychology
- ☐ Nursing
- ☐ Other *(please specify)*:

41. Do you have subspeciality training?

- ○ Yes
- ○ No

If yes, please specify

42. How many years have you worked as a consultant (after completion of your specialization)?

- <5 years
- 5-10 years
- 10-15 years
- 15-20 years
- >20 years

43. In which practice setting do you spend the majority of your clinical time?

- Solo practice
- Single speciality group
- Multispeciality group
- University-based group
- Health care centre
- Goverment hospital or clinic
- Other public or private hospital or clinical setting
- Student
- Retired
- Other *(please specify)*:

44. What is your age (optional)?

45. What is your gender (optional)?

- Male
- Female

46. From which organisation have you received the questionnaire?

- EADC
- EFNS
- IPA
- Other *(please specify)*:

Continue >

47. Please provide any additional comments on the topic of this survey
