# Supplementary material for: Use of mild cognitive impairment and prodromal AD/MCI due to AD in clinical care: a European survey
Source: Alzheimers Res Ther. 2019 Aug 22;11:74. doi: 10.1186/s13195-019-0525-9 (PMC6706888; doi:10.1186/s13195-019-0525-9)
Supplement: Supplementary file 2 — Figure S1. Counseling and medication prescription in patients with MCI. Figure S2. Benefits and drawbacks of MCI as clinical diagnosis. Figure S3. Counseling and medication prescription in patients with and without prodromal AD. (DOCX 572 kb) [file 13195_2019_525_MOESM2_ESM.docx]

Figure 1.1: Counseling and medication prescription in patients with MCI


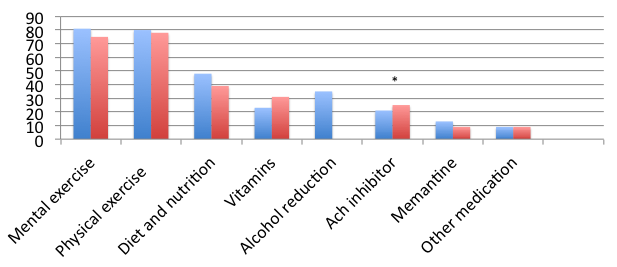


Frequency of response (%) in EAN/EADC survey (blue column) and AAN survey (red column) who routinely counsel on the above topics.

Differences frequencies between EAN/EADC and AAN members were tested using chi-square. *p<0.01

Figure 1.2: Benefits and drawbacks of MCI as clinical diagnosis

Blue bars are frequencies (in %)of respondents of EAN/EADC. Red bars are frequencies (in %) of respondents of the AAN survey. Frequency (%) of respondents that strongly or somewhat agreed on the above statements. Abbreviations: EAN, European Academy of Neurology; EADC, European Alzheimer’s Disease Consortium; AAN, American Academy of Neurology.

Figure 1.3: Counseling and medication prescription in patients with and without prodromal AD

Frequency (%) of respondents that routinely counseled or prescribed treatment in patients with ‘prodromal AD’ (blue column) or no prodromal AD’(red column). Differences frequencies between ‘prodromal AD’ and ‘ no prodromal AD’ were tested using chi-square. *p<0.01
